# Characteristics of SARS-CoV-2 variants of concern B.1.1.7, B.1.351 or P.1: data from seven EU/EEA countries, weeks 38/2020 to 10/2021

**DOI:** 10.2807/1560-7917.ES.2021.26.16.2100348

**Published:** 2021-04-22

**Authors:** Tjede Funk, Anastasia Pharris, Gianfranco Spiteri, Nick Bundle, Angeliki Melidou, Michael Carr, Gabriel Gonzalez, Alejandro Garcia-Leon, Fiona Crispie, Lois O’Connor, Niamh Murphy, Joël Mossong, Anne Vergison, Anke K. Wienecke-Baldacchino, Tamir Abdelrahman, Flavia Riccardo, Paola Stefanelli, Angela Di Martino, Antonino Bella, Alessandra Lo Presti, Pedro Casaca, Joana Moreno, Vítor Borges, Joana Isidro, Rita Ferreira, João Paulo Gomes, Liidia Dotsenko, Heleene Suija, Jevgenia Epstein, Olga Sadikova, Hanna Sepp, Niina Ikonen, Carita Savolainen-Kopra, Soile Blomqvist, Teemu Möttönen, Otto Helve, Joana Gomes-Dias, Cornelia Adlhoch, on behalf of COVID study groups

**Affiliations:** 1 European Centre for Disease Prevention and Control (ECDC), Stockholm, Sweden; 2 National Virus Reference Laboratory (NVRL), University College Dublin, Dublin, Ireland; 3 International Collaboration Unit, Research Center for Zoonosis Control, Hokkaido University, Sapporo, Japan; 4 Centre for Experimental Pathogen Host Research, University College Dublin, Dublin, Ireland on behalf of the All Ireland Infectious Diseases (AIID) Cohort; 5 Teagasc Food Research Centre, Moorepark, Fermoy, Ireland on behalf of the Irish Coronavirus Sequencing Consortium (ICSC); 6 Health Service Executive - Health Protection Surveillance Centre (HPSC), Dublin, Ireland; 7 Health Directorate, Findel, Luxembourg; 8 National Health Laboratory, Dudelange, Luxembourg; 9 Istituto Superiore di Sanità, Rome, Italy; 10 Directorate of Analysis and Information, Directorate-General of Health, Lisbon, Portugal; 11 Bioinformatics Unit, Infectious Diseases Department, National Institute of Health Dr. Ricardo Jorge, Lisbon, Portugal; 12 Health Board, Tallinn, Estonia; 13 Finnish Institute for Health and Welfare (THL), Department of Health Security, Helsinki, Finland; 14 The COVID study groups are listed at the end of this article under Acknowledgements

**Keywords:** Europe, SARS-CoV-2, surveillance, variants of concern, COVID-19

## Abstract

We compared 19,207 cases of SARS-CoV-2 variant B.1.1.7/S gene target failure (SGTF), 436 B.1.351 and 352 P.1 to non-variant cases reported by seven European countries. COVID-19 cases with these variants had significantly higher adjusted odds ratios for hospitalisation (B.1.1.7/SGTF: 1.7, 95% confidence interval (CI): 1.0–2.9; B.1.351: 3.6, 95% CI: 2.1–6.2; P.1: 2.6, 95% CI: 1.4–4.8) and B.1.1.7/SGTF and P.1 cases also for intensive care admission (B.1.1.7/SGTF: 2.3, 95% CI: 1.4–3.5; P.1: 2.2, 95% CI: 1.7–2.8).

Here, we analyse coronavirus disease (COVID-19) cases infected with any of the three severe acute respiratory syndrome coronavirus 2 (SARS-CoV-2) variants of concern (VOC): B.1.1.7/S gene target failure (SGTF), B.1.351 or P.1. We compare them with cases reported as infected with non-VOC virus with a focus on disease severity.

## SARS-CoV-2 variant viruses

In December 2020, the United Kingdom (UK) reported an emerging SARS-CoV-2 VOC classified as Pangolin lineage B.1.1.7 [1]. In the UK, and shortly thereafter in Denmark, B.1.1.7 infections increased rapidly. In parallel to the identification of B.1.1.7, increased whole-genome sequencing (WGS) efforts globally led to the identification of further SARS-CoV-2 VOC, including B.1.351 (described in South Africa) or P.1 (originating in Brazil) [2-6]. While viral evolution is expected and has occurred since the discovery of SARS-CoV-2, these VOC were associated with higher transmissibility and severity as well as altered antigenicity with potential implications for acquired immunity or effectiveness of current vaccines compared with other circulating lineages lacking particular defining mutations such as E484K, N501Y or del69-70 [7-12].

## Reporting of SARS-CoV-2 variants in the EU/EEA

On a weekly basis, countries in the European Union and European Economic Area (EU/EEA) report data on COVID-19 cases to The European Surveillance System (TESSy) hosted at the European Centre for Disease Prevention and Control (ECDC). In response to the emerging VOC, ECDC implemented new reporting variables for variants on 24 December 2020 allowing retrospective data upload (Supplement A). COVID-19 cases that do not have the VOC-defining mutations should be reported as non-VOC because of the complexity of the taxonomy of SARS-CoV-2. However, countries sequence only a minor proportion of all SARS-CoV-2 positive specimens (Supplement A, Figures S1 and S2 [6]).

We analysed data on COVID-19 cases infected with SARS-CoV-2 VOC (below referred to as B.1.1.7/SGTF, B.1.351 and P.1 cases) reported to TESSy for weeks 38/2020 to 10/2021 by seven countries (Cyprus, Estonia, Finland, Ireland, Italy, Luxembourg and Portugal) (Figure). Data included information on sex, age, clinical symptoms, pre-existing conditions, hospital and intensive care unit (ICU) admission and outcome (i.e. survived or died). The spike (S) gene deletion (del 69–70) is present in multiple lineages including B.1.1.7 and has been used for rapid screening using qRT-PCR (SGTF) because of a strong correlation between B.1.1.7 cases and SGTF [13,14]. Portugal showed a > 90% correlation between SGTF and B.1.1.7 cases and almost all (98%) of the SGTF cases included in this analysis came from Portugal (Figure). We therefore considered all SGTF cases as B.1.1.7 in our analysis [15].

**Figure:**
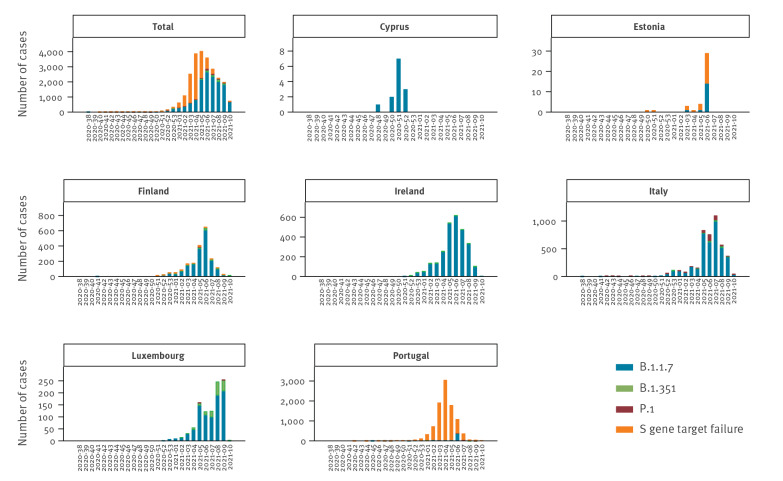
Reported SARS-CoV-2 VOC cases, by reporting country and week of reporting, EU/EEA, weeks 38/2020–10/2021 (n = 23,343)

EEA: European Economic Area; EU: European Union; SARS-CoV-2: severe acute respiratory syndrome coronavirus 2; VOC: Variant of concern.

 We compared VOC cases caused by variants B.1.1.7/SGTF, B.1.351 or P.1 to non-VOC cases which derived from the same surveillance system (Supplement A). Cases reported with missing or unknown information on the virus variant were excluded because an increasing number of VOC cases with missing confirmation would be included in this group and introduce a bias when used as a reference category (Supplement A, Table S1 and Figure S3). 

Proportions, medians and means were calculated and compared using chi-squared, rank sum and t-tests with a significance of p = 0.05 using STATA v16.1. Different logistic regression models, 1:1 matched (on 10-year age groups, sex and week of reporting, using conditional logistic regression) and unmatched (adjusted for age, sex, week and country, including having a pre-existing condition and healthcare worker status, using logistic regression), were applied to assess differences in severity (hospitalisation, ICU and death) between VOC (B.1.1.7/SGTF, B.1.351 and P.1) and non-VOC cases.

## Characteristics of SARS-CoV-2 variants of concern

Of 3.2 million COVID-19 cases reported from the included countries during the study period (Supplements A and B), 23,343 had information on SARS-CoV-2 variants, of which 19,995 were VOC and 3,348 non-VOC cases included in this analysis. Among all cases with information on the variant, B.1.1.7/SGTF was the most frequently reported VOC (19,207; 82.3%), followed by B.1.351 (436; 1.9%) and P.1 (352; 1.5%) (Table 1). Cases from Portugal accounted for almost half (9,740) of the reported VOC. VOC cases have been increasing since week 51/2020, with lower numbers in weeks 6 to 10 probably caused by reporting delay (Figure). The male to female ratio differed slightly between the variants, ranging from 49% to 54% of male cases (p < 0.001) (Table 1).

**Table 1:** Characteristics of reported SARS-CoV-2 VOC and non-VOC cases, EU/EEA, weeks 38/2020–10/2021 (n = 23,343)

Characteristics	B.1.1.7/SGTF	%	B.1.351	%	P.1		%	non-VOC	%
Total^a^	19,207		436		352			3,348	
Sex									
Female	9,700	50.5	211	48.4	179		50.9	1,541	46
Male	9,506	49.5*	225	51.6	173		49.1	1,807	54
Total^b^	19,206		436		352			3,348	
Age (years)									
Range	0–103		0–109		2–101			0–105	
Median	39		42		46			38	
Mean	39		43		46*			40	
Standard deviation	21		22		25			21	
Age group (years)									
0–19	3,730	19.4	60	13.8	79		22.4	569	17.0
20–39	6,005	31.3	147	33.7	66		18.8	1,195	35.7
40–59	6,151	32.0	139	31.9	107		30.4	986	29.5
60–79	2,538	13.2	62	14.2	58		16.5	390	11.6
≥ 80	783	4.1	28	6.4	42		11.9	208	6.2
Total^b^	19,207		436		352			3,348	
Symptoms									
No	2,025	27.4	3	9.7	2		33.3	125	18.6
Yes	5,365	72.6*	28	90.3	4		66.7	547	81.4
Total^b^	7,390		31		6			672	
Pre-existing condition									
No	10,608	55.2	89	20.4	254		72.2	369	11.0
Yes	8,599	44.8*	347	79.6*	98		27.8*	2,979	89.0
Total^b^	19,207		436		352			3,348	
Hospitalisation									
No	7,855	89.0	309	80.7	272		80.0	2,399	92.5
Yes	966	11.0*	74	19.3*	68		20.0*	195	7.5
Total^b^	8,821		383		340			2,594	
ICU admission									
No	8,593	98.6	380	97.7		332	97.9	2,553	99.4
Yes	121	1.4*	9	2.3*		7	2.1*	16	0.6
Total^b^	8,714		389			339		2,569	
Mortality/outcome									
Alive/ on treatment	7,490	98.0	309	94.8		295	96.1	1,773	96.0
Died	155	2.0*	17	5.2		12	3.9	73	4.0
Total^b^	7,645		326			307		1,846	
Cases imported									
No	6,143	98.5	107	91.5	263		98.5	694	99.6
Yes	93	1.5*	10	8.5*	4		1.5	3	0.4
Total^b^	6,236		117		267			697	
Healthcare worker									
No	11,985	94.3	358	92.7	85		80.2	2,425	92.0
Yes	730	5.7*	28	7.3	21		19.8*	211	8.0
Total^b^	12,715		386		106			2,636	

EEA: European Economic Area; EU: European Union; ICU: intensive care unit; SARS-CoV-2: severe acute respiratory syndrome coronavirus 2; SGTF: S gene target failure; VOC: variant of concern.

^a ^Total number of cases included in the analysis.

^b ^Total number of cases for whom information was reported.

Totals for all included cases are given on top; totals for the individual characteristics refer to the cases for whom this information was reported.

* p < 0.05 compared with non-VOC cases (chi-squared or t-test).

The proportion of B.1.1.7/SGTF cases in the oldest age group decreased slightly over the reporting weeks (Supplement A, Figure S4). Our analysis showed that the proportion of cases in younger age groups (< 60 years) was similar for VOC and non-VOC cases, with similar mean ages for B.1.1.7/SGTF, B.1.351 and non-VOC but significantly older mean age for P.1 cases (Table 1).

Among the VOC cases with available information, the majority were domestic cases, with 1.5% of B.1.1.7/SGTF and P.1 cases and 8.5% of B.1.351 cases reported as importations, compared with 0.4% of non-VOC cases (Table 1). Healthcare workers were slightly less represented among VOC cases than among non-VOC cases. The exception was P.1 with 19.8% of cases being a healthcare worker (Table 1). No COVID-19 case infected with a VOC was reported as pregnant.

Among the B.1.1.7/SGTF cases, 72.6% (5,365/7,390) were reported symptomatic, fewer than among the non-VOC cases (81.4%; 547/672; p < 0.001), which in turn was lower than the proportion of symptomatic B.1.351 cases (90.3%; 28/31; p = 0.2; Table 1, Supplement A, Figure S5). Cases of infection with P.1 with available information on this variable were too rare to allow a comparison with the other groups. The proportion of cases who reported any pre-existing condition was significantly lower among B.1.1.7/SGTF, B.1.351 and P.1 than among non-VOC cases (p < 0.001; Table 1). The lower likelihood of having pre-existing conditions was confirmed in the matched analysis for all VOC cases, with an adjusted odds ratio (aOR) of 0.08 (95% confidence interval (CI): 0.07–0.1) for B.1.1.7/SGTF, an aOR of 0.57 (95% CI: 0.38–0.86) for B.1.351 and an aOR of 0.02 (95% CI: 0.01–0.06) for P.1 compared with non-VOC cases.

## Differences in severity between VOC and non-VOC cases

A larger proportion of VOC cases were admitted to hospital (B.1.1.7/SGTF 11.0%; B.1.351 19.3%, and P.1 20.0%; p < 0.001 for all VOC) and ICU (B.1.1.7/SGTF 1.4%, p = 0.002; B.1.351 2.3%, p = 0.001 and P.1 2.1%, p = 0.005) compared with non-VOC cases (7.5%, hospitalised and 0.6% requiring ICU; Table 1, Supplement A, Figure S6). Of all hospitalisations with any VOC, 58.3% were male (646/1,108), which was comparable to non-VOC cases (55.4% male; p = 0.4). Hospitalised B.1.1.7/SGTF cases were significantly younger (mean age: 63 years, median age: 65 years; p < 0.001) than non-VOC (mean: 69 years, median: 75 years) in contrast to B.1.351 (mean and median age: 67 years; p = 0.1) and P.1 cases (mean: 71 years, median: 76 years; p = 0.7) which were of a similar age as the non-VOC cases.

Both the matched and unmatched multivariable analysis found that B.1.1.7/SGTF, B.1.351 and P.1 cases had significantly higher odds of hospitalisation than non-VOC cases (aOR: 1.6–4.2 (matched) vs 1.7–3.6 (unmatched)) (Table 2). In the unmatched analysis, B.1.1.7/SGTF, B.1.351 or P.1 cases were, respectively, 2.3, 3.3 and 2.2 times more likely to be admitted to ICU than non-VOC cases.

**Table 2:** Logistic regression for outcome admission to hospital or intensive care unit for cases with SARS-CoV-2 VOC B.1.1.7/SGTF, B.1.351 and P.1 compared with non-VOC cases, EU/EEA, weeks 38/2020–10/2021 (n = 23,342)

VOC	Hospitalisation						Intensive care unit admission						Death					
	Matched			Multivariable			Matched			Multivariable			Matched			Multivariable		
	Cases	aOR^a^	95% CI	Cases	aOR^b^	95% CI	Cases	aOR^a^	95% CI	Cases	aOR^b^	95% CI	Cases	aOR^a^	95% CI	Cases	aOR^b^	95% CI
B.1.1.7/SGTF^c^	300	1.6	1.2–2.3	11,414	1.7	1.0–2.9	48	1.2	0.5–2.6	11,282	2.3	1.4–3.5	64	0.5	0.3–1.1	9,490	0.5	0.3–0.9
B.1.351	112	3.7	1.9–6.9	2,977	3.6	2.1–6.2	12	2.0	0.4–10.9	2,958	3.3	1.9–5.7	30	1.1	0.4–3.2	2,172	1.1	0.4–3.4
P.1	104	4.2	2.1–8.4	2,934	2.6	1.4–4.8	14	6.0	0.7–49.8	2,908	2.2	1.8–2.9	NR			2,153	0.6	0.3–1.0

aOR: adjusted odds ratio; CI: confidence interval; EEA: European Economic Area; EU: European Union; NR: no result; SARS-CoV-2: severe acute respiratory syndrome coronavirus 2; SGTF: S gene target failure; VOC: variant of concern.

^a^ Conditional logistic regression of 1:1 matched (age groups at 10-year increments, sex and week of reporting).

^b^ Adjusted for age, sex, week of reporting and country.

^c^ Excluding one case with missing information on sex.

The columns ’Cases’ represent all respective VOC and non-VOC cases included in the analysis.

In the age-stratified models, B.1.1.7/SGTF cases in the age groups 20–39 and 40–59 years had, respectively, 3.0 and 2.3 times higher odds of hospitalisation when compared with non-VOC cases, while ICU admission or death did not differ significantly in any age group (Table 3). For B.1.351 cases, we observed 3.5–3.6 times higher odds of hospitalisation for age groups 40–59 and 60–79 years compared with non-VOC cases of the same age. Admission to ICU was significantly more likely for B.1.351 cases (aOR: 8; 95% CI: 3.7–17.3) aged 40–59 years. For P.1 cases, we observed between 3.0 and 13.1 times higher odds of hospitalisation in the age groups 20–39, 40–59 and 60–79 as well as a 2.9–13.9 times higher odds of ICU admission (40–59, 60–79 and ≥ 80 age groups).

**Table 3:** Logistic regression for hospital and intensive care unit admission or death for cases with SARS-CoV-2 VOC B.1.1.7/SGTF B.1.351 and P.1 compared with non-VOC cases, EU/EEA, weeks 38/2020–10/2021 (n = 23,343)

VOC and age group	Hospitalisation		ICU admission		Death	
	aOR^a^	95% CI	aOR^a^	95% CI	aOR^a^	95% CI
**B.1.1.7/SGTF**						
**0–19 years**	**n = 1,860**		**n = 121**		**NR**	
B.1.1.7/SGTF	1.0	0.4–2.7	1	Omitted		
Pre-existing condition	1.0	0.2–4.3	1	Omitted		
Healthcare worker	1.0	Omitted	1	Omitted	Not included	
**20–39 years**	**n = 3,167**		**n = 2,642**		**NR**	
B.1.1.7/SGTF	3.0*	1.4–6.8	1.0	Omitted		
Pre-existing condition	0.5	0.2–1.6	0.5	0.3–0.8		
Healthcare worker	1.1	0.5–2.7	Not included		Not included	
**40–59 years**	**n = 3,017**		**n = 3,511**		**n = 2,546**	
B.1.1.7/SGTF	2.3*	1.0–5.4	2.1*	1.0–4.7	0.3	0.1–0.8
Pre-existing condition	0.7	0.1–3.1	5.4*	1.0–29.9	1.0	Omitted
Healthcare worker	0.4	0.2–0.8	Not included		Not included	
**60–79 years**	**n = 1,263**		**n = 1,490**		**n = 1,338**	
B.1.1.7/SGTF	1.7	0.9–3.4	1.7	0.8–3.8	0.7	0.4–1.2
Pre-existing condition	0.4	0.1–1.5	0.8	0.4–1.5	2.4	0.9–6.4
Healthcare worker	0.3	0.1–0.9	Not included		Not included	
**≥ 80 years**	**n = 565**		**n = 612**		**n = 526**	
B.1.1.7/SGTF	1.2	0.6–2.3	1.1	0.3–4.2	0.4	0.2–1.0
Pre-existing condition	0.2	0.0–3.3	0.4	0.0–16.3	1.4	0.9–2.0
Healthcare worker	1.0	Omitted	Not included		Not included	
**B.1.351**						
**0–19 years**	**n = 504**		**NR**		**NR **	
B.1.351	2.5	0.7–9.1				
Pre-existing condition	0.4	0.1–1.2				
Healthcare worker	1.0	Omitted	Not included		Not included	
**20–39 years**	**n = 894**		**NR**		**NR**	
B.1.351	3.0	0.7–12.4				
Pre-existing condition	2.8	0.7–11.6				
Healthcare worker	1.0	Omitted	Not included		Not included	
**40–59 years**	**n = 869**		**n = 398**		**n = 442**	
B.1.351	3.5*	2.5–5.1	8.0*	3.7–17.3	1.0	Omitted
Pre-existing condition	0.7	0.3–1.6	1.0	Omitted	1.0	Omitted
Healthcare worker	0.2	0.0–2.0	Not included		Not included	
**60–79 years**	**n = 337**		**n = 313**		**n = 236**	
B.1.351	3.6*	1.1–11.9	2.0	0.7–6.0	1.8	0.7–4.8
Pre-existing condition	0.7	0.4–1.5	1.0	Omitted	1.0	Omitted
Healthcare worker	0.2	0.0–1.1	Not included		Not included	
**≥ 80 years**	**n = 215**		**n = 198**		**n = 168**	
B.1.351	4.1	0.8–20.4	4.3	0.6–33.1	1.0	0.3–2.9
Pre-existing condition	1.0	Omitted	1.0	Omitted	1.0	Omitted
Healthcare worker	1.0	Omitted	Not included		Not included	
**P.1**						
**0–19 years**	**n = 453**		**NR**		**NR**	
P.1	1.0	Omitted				
Pre-existing condition	0.6	0.2–2.2				
Healthcare worker	1.0	Omitted	Not included		Not included	
**20–39 years**	**n = 811**		**NR **		**NR **	
P.1	13.1*	6.5–26.5				
Pre-existing condition	2.7	1.2–6.1				
Healthcare worker	1.0	Omitted	Not included		Not included	
**40–59 years**	**n = 787**		**n = 363**		**n = 442**	
P.1	3.0*	1.5–5.8	6.8*	2.4–19.6	1.0	Omitted
Pre-existing condition	0.5	0.2–1.0	1.0	Omitted	1.0	Omitted
Healthcare worker	0.1	0.0–1.7	Not included		Not included	
**60–79 years**	**n = 294**		**n = 340**		**n = 267**	
P.1	3.7*	1.9–7.0	2.9*	1.6–5.4	1.6	0.8–3.2
Pre-existing condition	0.9	0.5–1.6	1.5	0.6–4.0	7.7	3.0–19.6
Healthcare worker	0.2	0.0–1.1	Not included		Not included	
**≥ 80 years**	**n = 191**		**n = 183**		**n = 176**	
P.1	1.5	0.8–2.6	13.9*	2.1–89.4	1.3	0.6–3.0
Pre-existing condition	1.0	Omitted	1.0	Omitted	3.7	3.4–4.0
Healthcare worker	1.0	Omitted	Not included		Not included	

aOR: adjusted odds ratio; CI: confidence interval; EEA: European Economic Area; EU: European Union; ICU: intensive care unit; NR: no result; SARS-CoV-2: severe acute respiratory syndrome coronavirus 2; SGTF: S gene target failure; VOC: variant of concern.

^a^ Adjusted for reporting country (seven clusters), sex and week of reporting.

* indicates significance (p < 0.05).

Analysis stratified by age group and adjusted for sex, country and week of reporting. n indicates the number of total cases included in the analysis for each age group (B.1.1.7/SGTF, B.1.351 or P.1 as well as non-VOC cases); Omitted variable, no 95% CI calculated.

A total of 184 (2.2%) deaths were reported among VOC cases; B.1.1.7/SGTF (n = 155), B.1.351 (n = 17), and P.1 cases (n = 12; Table 1), ranging in age between 41–99 years. The matched and multivariable analysis did not show increased risk of death.

## Ethical statement

Ethical approval was not required for this study, data are collected through national surveillance.

## Discussion

This analysis outlines the characteristics of SARS-CoV-2 VOC infections in seven EU/EEA countries and suggests a higher risk for hospitalisation, and also for ICU admission in age groups < 60 years for B.1.1.7/SGTF, B.1.351 and P.1. Similarly, Germany reported increased hospitalisation in age groups < 60 years following B.1.1.7 dominance [16]. Earlier, higher infection rates in younger, school-age age groups with subsequent infections across all age groups have been observed in the UK [7,9]. Higher odds of hospitalisation for B.1.1.7 cases have also been reported by Denmark [8], but there is currently a lack of published data on severity for B.1.351 and P.1.

Overall, only a minor proportion of all SARS-CoV-2-positive specimens are sequenced, however, both VOC and non-VOC cases presented in this analysis are derived from the same sampling frame. It is possible that sampling and sequencing were biased towards hospitalised cases, which could lead to an overestimation of the risk; however, this would be the same for non-VOC cases included in the analysis. Reporting of variant cases is likely to be affected by both underreporting and reporting delay, as WGS efforts take time (≥ 3 weeks), but countries are setting up systematic sampling for WGS monitoring of the circulation of emerging VOC (Supplement A) [17,18]. Supplement B presents a summary of B.1.1.7 and SGTF cases and of all COVID-19 cases including those with unknown or missing variant information as well as the logistic regression models comparing all non-VOC cases and those with missing/unknown information (possibly including unconfirmed B.1.1.7 cases), indicating also potential higher mortality associated with B.1.351 and P.1. Data were included until week 10/2021 and this may have been too soon after the introduction of these VOC into EU/EEA countries to detect higher mortality as observed in countries not included in this analysis for B.1.1.7 [8-10]. Also, information about hospitalisation, ICU admission and outcome may not have been available for the most recently infected cases. Although testing for variant viruses in December 2020 mainly targeted travellers from affected countries and their contacts, only a minority of cases in our analysis for whom data was available for the study period were reported as importations. Testing of contacts of travellers or targeted testing in schools or workplaces generally or in response to outbreaks could also explain higher detection rates in younger age groups. Finally, the pooling of SGTF cases with B.1.1.7 cases could have led to some misclassification, despite evidence of high correlation between these cases. However, a small minority of 130 such cases had information on hospitalisation, which makes it unlikely that they had substantially impacted the severity outcomes.

## Conclusion

We show an increased risk for hospitalisations and ICU admission associated with the SARS-CoV-2 variants B.1.1.7/SGTF, B.1.351 and P.1, also in middle-aged individuals, which underlines the necessity to rapidly reach high levels of vaccine coverage and adhere to public health measures to reduce SARS-CoV-2 incidence and prevent severe cases. Enhanced testing and contact tracing implemented with a special focus on cases with VOC are also measures to reduce spread.

## Supplementary Data

873000 SupplementA

## Supplementary Data

42000 SupplementB
