# Supplementary material for: Characteristics of SARS-CoV-2 variants of concern B.1.1.7, B.1.351 or P.1: data from seven EU/EEA countries, weeks 38/2020 to 10/2021
Source: Euro Surveill. 2021 Apr 22;26(16):2100348. doi: 10.2807/1560-7917.ES.2021.26.16.2100348 (PMC8063589; doi:10.2807/1560-7917.ES.2021.26.16.2100348)
Supplement: SupplementA [file 21-00348_ADLHOCH_SupplementA.pdf]

Supplement A, Online material:

This supplementary material is hosted by Eurosurveillance as supporting information alongside the article *Characteristics of SARS-CoV-2 variants of concern B.1.1.7, B.1.351 or P.1: data from seven EU/EEA countries, weeks 38/2020 to 10/2021*, on behalf of the authors, who remain responsible for the accuracy and appropriateness of the content. The same standards for ethics, copyright, attributions and permissions as for the article apply. Supplements are not edited by Eurosurveillance and the journal is not responsible for the maintenance of any links or email addresses provided therein.

Supplement 1: The following definitions for reporting variant types have been added to The European Surveillance System (TESSy):

P.1 = Brazil variant; mutations: L18F, T20N, P26S, D138Y, R190S, K417T, E484K, N501Y, H655Y, T1027I, V1176F)

VOC_202012_01 = UK variant; mutations: del69-70, del144, N501Y, A570D, D614G, P681H, T716I, S982A, D1118H

501_V2 = SA variant; mutations: D80A, D215G, E484K, N501Y, A701V (and possibly L18F, del 242-244, R246I, K417N)

S_GENE_DELETION = Variant with deletion in S-gene; mutation: del 69-70 or negative S-gene RT-PCR

CLUSTER_5 = DK cluster 5. Defined by mutations: del 69-70, Y453F, I692V, M1229I

Y453F = Y453F associated with farmed minks; defined by mutation: Y453F

VARIANT_OTHER = Novel variant of potential concern. Provide details in VirusVariantOther

WILD_TYPE = None of the variants described for this variable

UNK = Sequence information unknown or not available

COVID-19 case with a variant virus of SARS-CoV-2 according to mutation pattern of specific concern identified by sequence analysis of the case, or in some cases by a specific RT-PCR pattern. If several apply, choose the most specific variant (highest number of matching mutations).

Supplement 2: Testing and reporting systems in the countries:

<u>Finland:</u>

In Finland we test everyone with any suitable symptoms for Covid-19 and also screen asymptomatic exposed people in the context of contact tracing and border surveillance upon entry.

From January 2020 to December 2020 we sequenced a sample of all positive cases mainly for research purposes (no variant findings). We started extensive variant screening and sequencing in week 51, December 2020 upon variant findings in Europe. The first findings were from entry screening and their contacts. We also screened contacts of known variant cases in transmission chains. From February 2021 onwards, we have sequenced a random sample of all positive cases throughout the country and also positive cases from entry screening and contacts of known variant cases (not all).

We report the sequencing data to Tessy according to the ECDC algorithm. We aim to submit all sequences to GISAID including those of non-variant cases, this is pending.

Ireland:

Real-time PCR testing is performed on all symptomatic cases and a subset of samples with Ct values ≤25 are selected for sequencing, being representative of age range and geographical distribution of the COVID-19 positive cases in the country. A small percentage of samples are sequenced based on SGD and N501Y or E484K mutation-specific PCRs. All variants of concern and variants of interest are reported to public health for enhanced action and to TESSy.

Italy

Italy has implemented a thorough testing strategy amid rapidly increased its testing capacity. The current national weekly average of testing is over 3600 swab tests per 100,000 inhabitants per week with 6.6% of positive tests.

Sequencing is recommended in all cases of confirmed SARS-CoV-2 infection: from countries with high VOC SARS-CoV-2 variant circulation, epidemiologically linked to cases with VOC SARS-CoV-2 infections, with S-gene target failure and in cases of reinfection or of vaccination failure.

Regional reference laboratories coordinate sequencing activities in their catchment area under the overall coordination of the National Reference Laboratory in ISS, Rome. Reporting is performed in the National Integrated surveillance platform for COVID-19 that combines epidemiological and basic microbiological metadata, this data is regularly sent to TESSy.

Luxembourg

The national reference laboratory for acute respiratory infections at LNS implements the following weekly sequencing activities for SARS-CoV2:

1) Sequencing specimens from all hospitalized positive cases

2) Sequencing specimens from all positive cases from Airport testing program

3) Sequencing specimens from all outbreaks and identified clusters

4) Systematic sequencing of specimens from reinfections and post-vaccination-infections

5) Population sequencing of specimens from representative regions and age groups, to follow the evolution of the different variants in the Luxembourg population.

All positive SARS-CoV-2 samples are referred from diagnostic laboratories nationwide to LNS where primary samples are extracted and processed for RT-PCR to select the eligible samples for sequencing (criterion: CT value < or = 35 are sequenced). Results of successfully sequenced samples are reported

to the health inspection and referring hospitals. Variant surveillance is done by amplicon based whole genome sequencing on an Illumina platform (to be extended by variant specific RT-PCR). The sequence coverage of all positive cases is on average 30%. Sequencing results are integrated in the contact tracing database hosted by the health inspection to follow up particular clusters (e.g. nursing homes, schools). Pseudonymised data is sent via Tessy to ECDC on a weekly basis.

Portugal

Portugal performs monthly sequencing nationwide surveys. In parallel, the Portuguese NIH continuously sequences suspected samples of: 1) VOCs (signalled by SNP assays and/or travel-history); 2) potential vaccine failures; 3) potential re-infection, etc. So far, this targeted sequencing usually accounts for less than 3% of all monthly sequenced samples. All sequences are deposited in GISAID and reported to TESSy.

Figure S1. Total number of sequences by data source per week

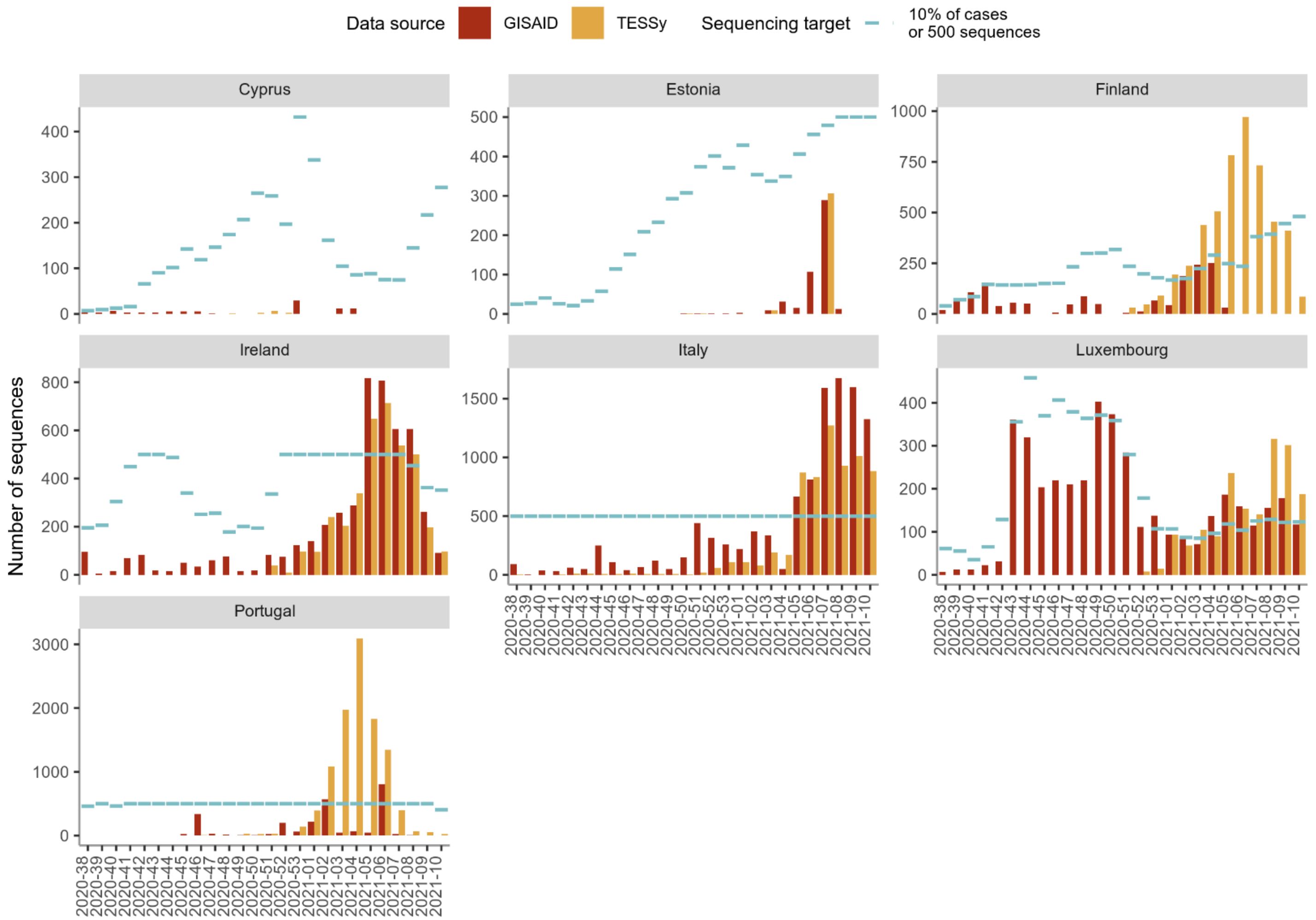

This figure bases on data submitted to TESSy up to week 2021-12 and extracted from GISAID EpiCoV database (https://www.gisaid.org/) by 30 March 2021. As the data extraction was done later than the rest of the analysis, slight differences to what was used for the analysis may exist due to retrospective data updates by countries.

Figure S2. Weekly variant distribution by country and data source (Data shown from the source with highest number of sequences in the period. Estimates only shown for weeks with a valid denominator)

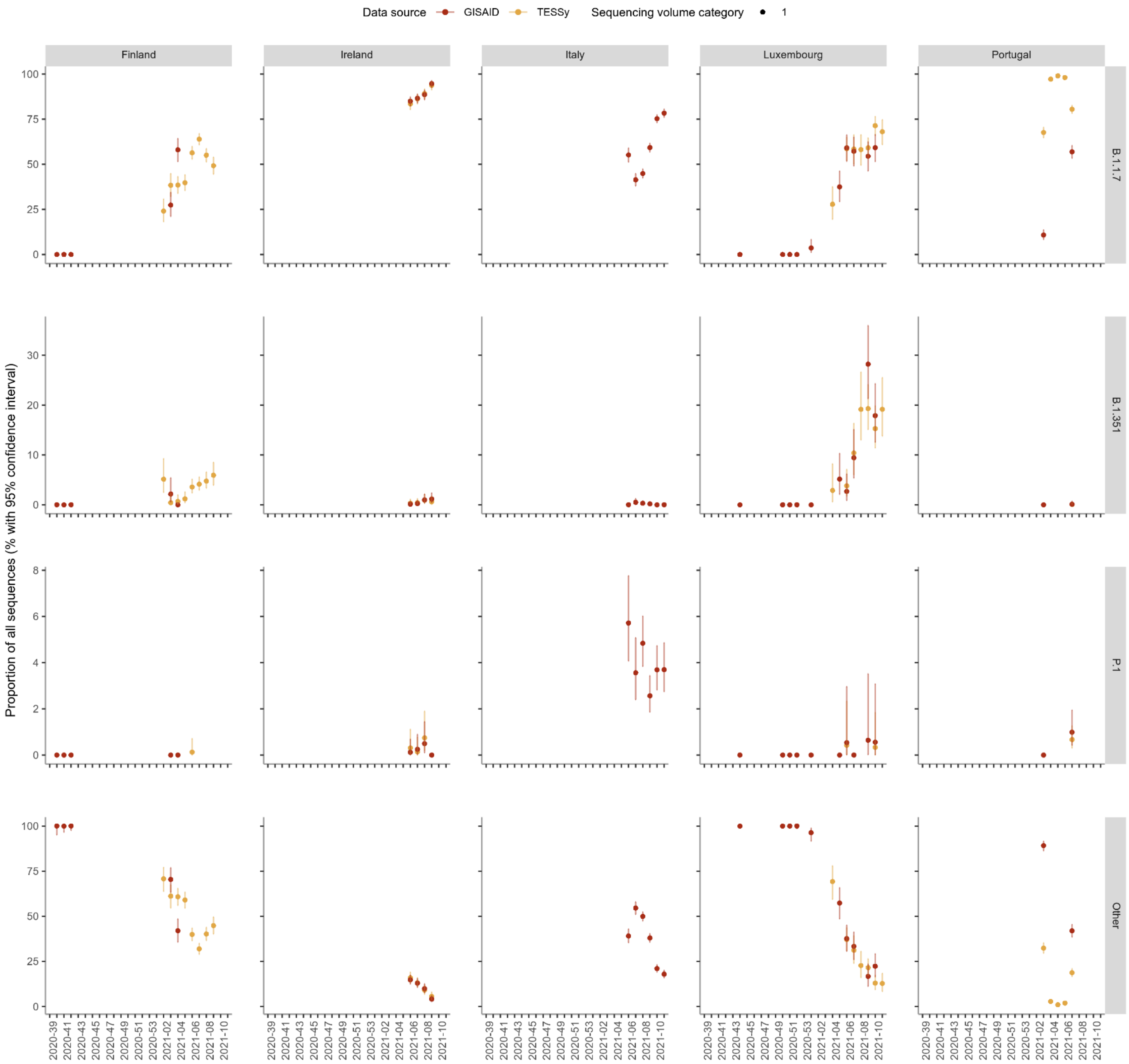

Note: Estimates only shown for weeks and data source in which the weekly sequencing volume was at least 500 sequences or 10% of all cases (this excludes Cyprus and Estonia). As not all generated sequences are reported there may be under-estimation of the sequencing activities in some countries. This figure bases on data submitted to TESSy up to week 2021-12 and extracted from GISAID EpiCoV database (https://www.gisaid.org/) by 30 March 2021. As the data extraction was done later than the rest of the analysis, slight differences to what was used for the analysis may exist due to retrospective data updates by countries.

Figure S3. Reported SARS-CoV-2 cases with unknown or missing variant type by reporting country and reporting week, EU/EEA, weeks 38/2020-10/2021

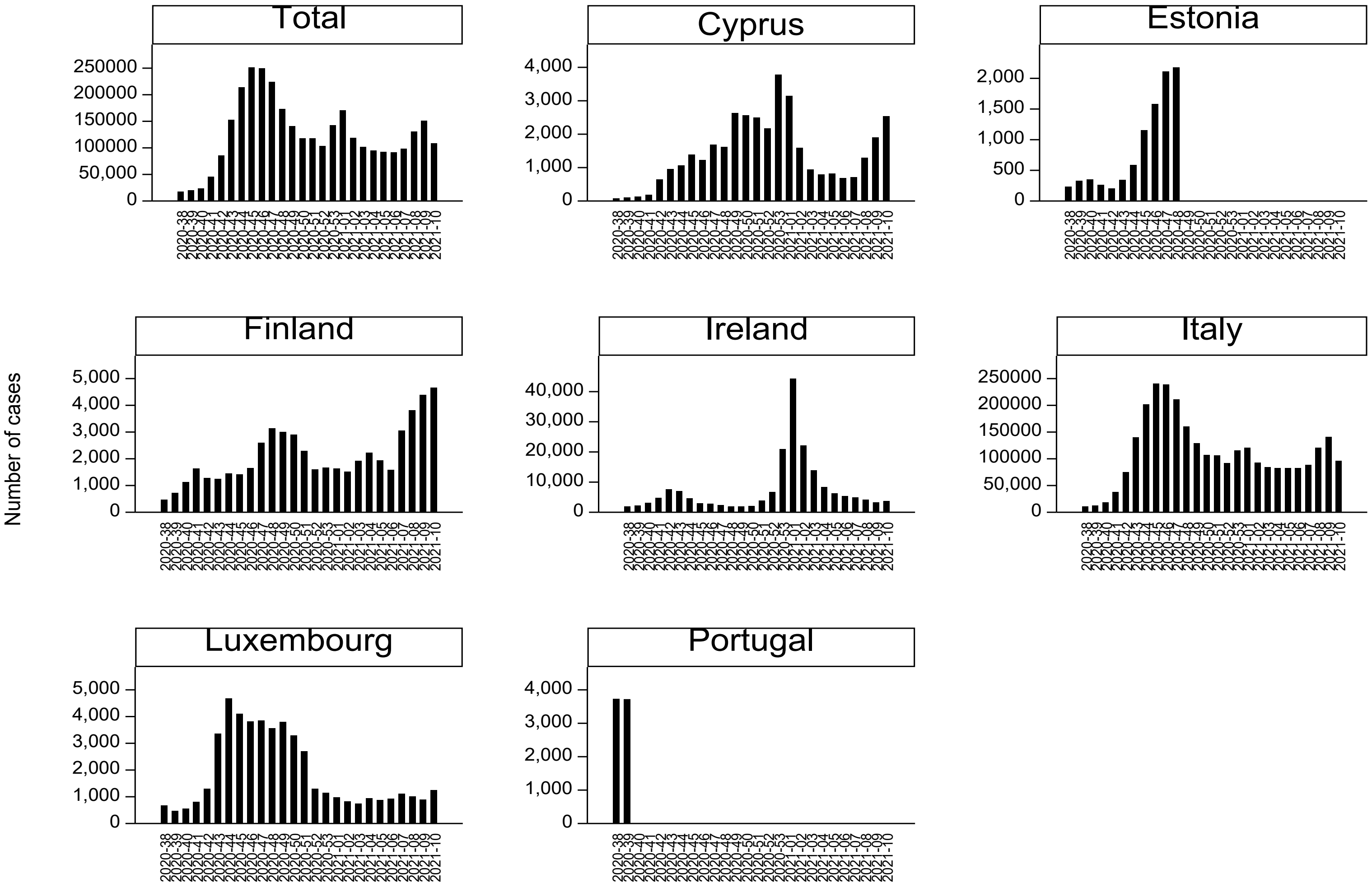

Figure S4. Proportion of SARS-CoV-2 cases by variant B.1.1.7/SGTF (n=19,207), B.1.351 (n=435), P.1 (n= 352) or non-VOC (n= 3,348) by age-group and week of reporting, seven EU/EEA countries, weeks 38/2020-10/2021

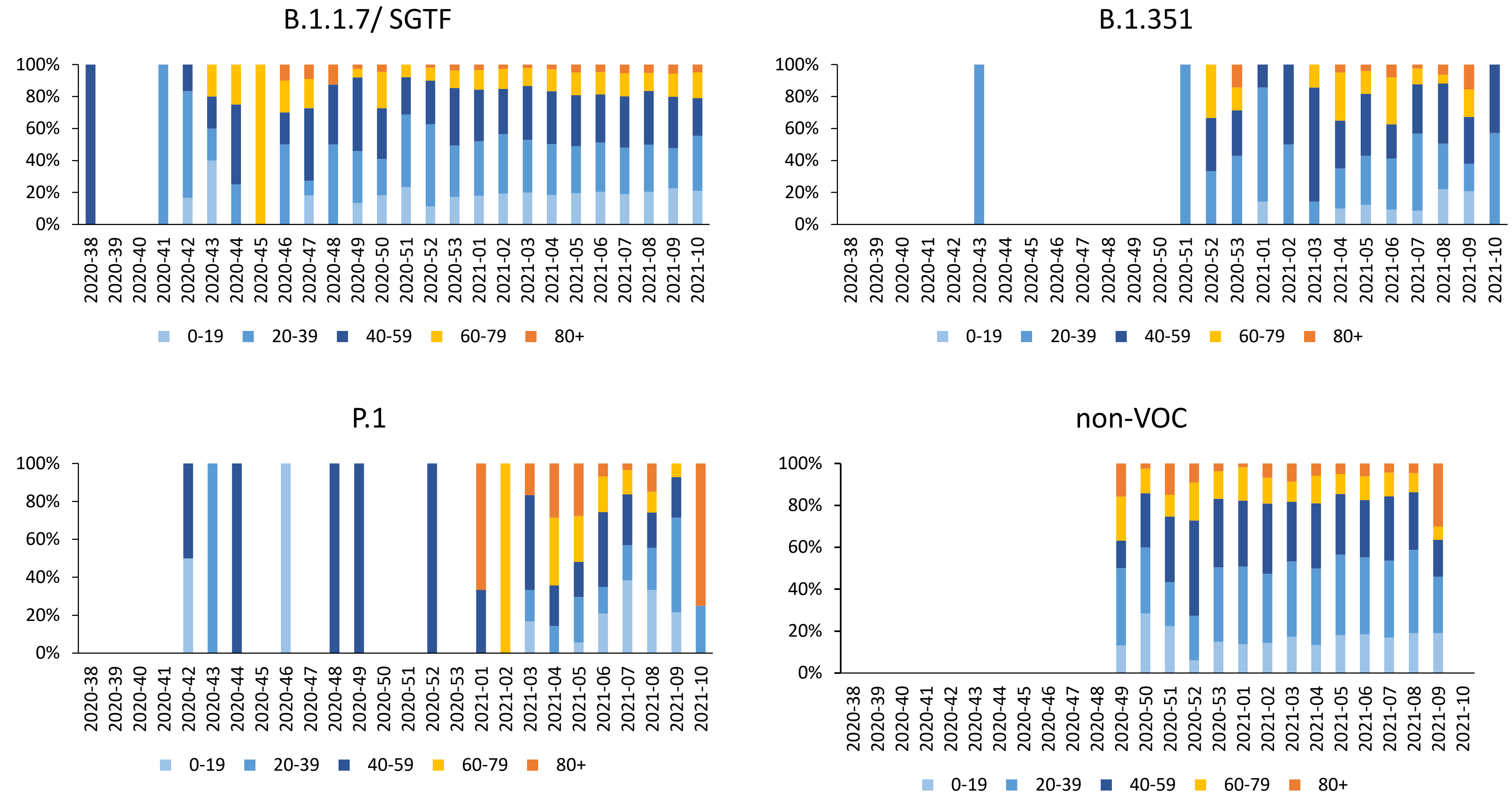

SGTF: S gene target failure; VOC: variant of concern

Figure S5. Proportion of cases with clinical symptoms and preconditions by virus variants B.1.1.7/SGTF, B.1.351, or non-VOC, EU/EEA, weeks 38/2020-10/2021

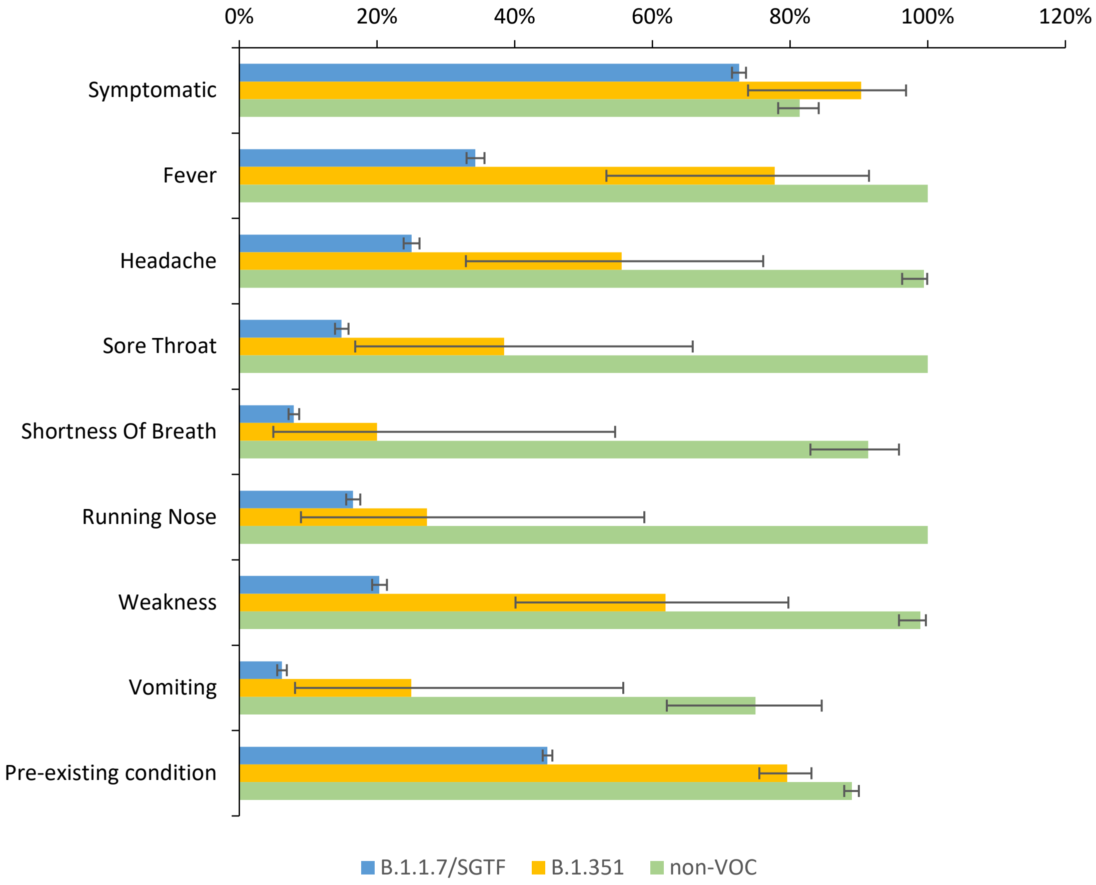

SGTF: S gene target failure; VOC: variant of concern

Figure S6. Proportion of cases admitted to hospital, intensive care or died following infection by B.1.1.7/SGTF, B.1.351, P.1 or non-VOC, EU/EEA, weeks 38/2020-10/2021

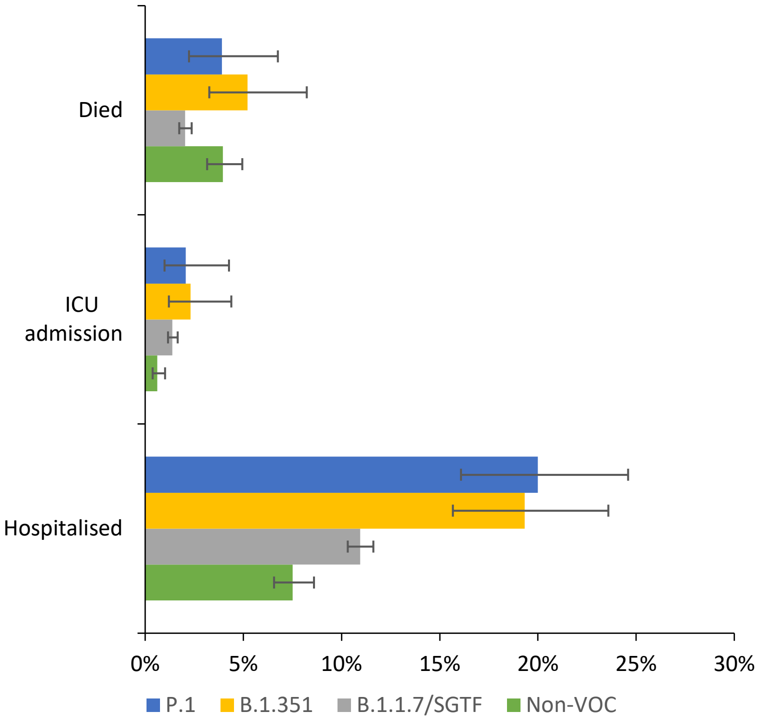

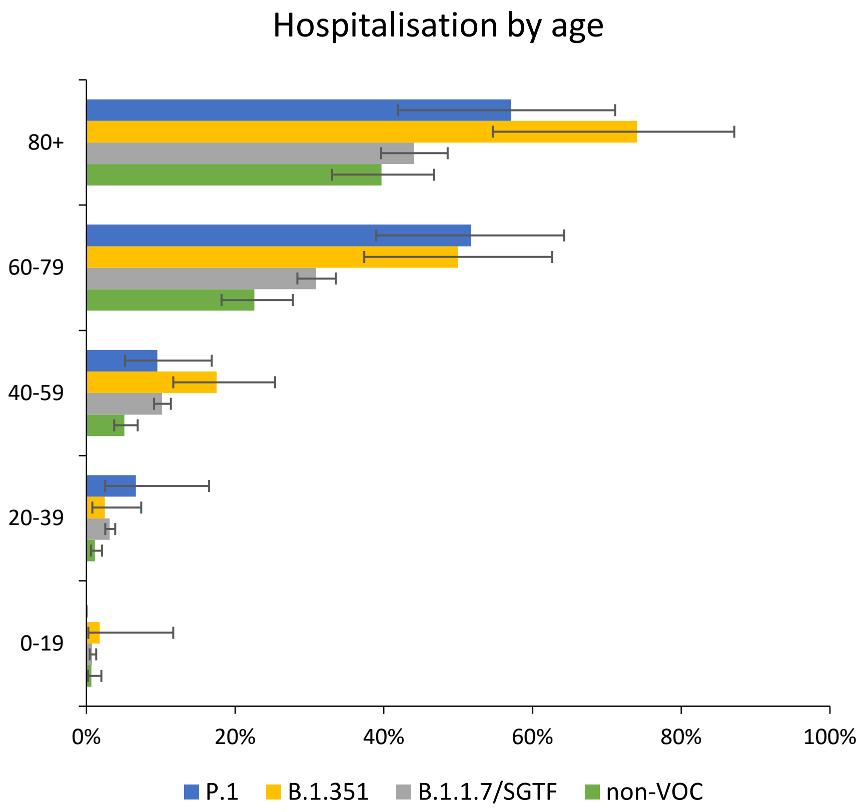

SGTF: S gene target failure; VOC: variant of concern

Supplement Table 1:

	B.1.1.7	%	SGTF	%	B.1.1.7/SGTF combined	%	P.1	%	B.1.351	%	non-VOC	%	Sample	Unknown/ missing	%
**Country**															
Cyprus	13		0		13		0		0		0		13	37,108	
Estonia	16		23		39		0		0		129		168	9,330	
Finland	1,778		126		1,904		1		129		1,808		3,842	54,926	
Ireland	2,679		0		2,679		0		24		637		3,340	192,813	
Italy	3,997		0		3,997		337		98		0		4,432	2,885,507	
Luxembourg	856		0		856		2		176		774		1,808	48,958	
Portugal	665		9,054		9,719		12		9		0		9,740	7,450	
Total	10,004	42.9	9,203	39.4	19,207	82.3	352	1.5	436	1.9	3,348	14.3	23,343	3,236,092	
**Sex**															
Female	4,905	49.0	4,795	52.1	9,700	50.5	179	50.9	211	48.4	1,541	46	11,631	1,657,106	51.2
Male	5,098	51.0	4,408	47.9	9,506	49.5	173	49.1	225	51.6	1,807	54	11,711	1,578,770	48.8
Total	10,003		9,203		19,207		352		436		3,348		23,342	3,235,876	
**Age**															
Range					0-103		2-101		0-109		0-105				
Mean	40		38		39		46		43		40		40	45	
Media	40		39		39		46		42		38		39	45	
Standard deviation	22		20		21		25		22		21		21	22	
**Age-group (years)**															
0-19	1,903	9.9	1,827	9.5	3,730	19.4	79	22.4	60	13.8	569	17.0	4,438	486,052	15.0
20-39	3,084	16.1	2,921	15.2	6,005	31.3	66	18.8	147	33.7	1,195	35.7	7,413	845,663	26.1
40-59	3,093	16.1	3,058	15.9	6,151	32.0	107	30.4	139	31.9	986	29.5	7,383	1,079,388	33.4
60-79	1,389	7.2	1,149	6.0	2,538	13.2	58	16.5	62	14.2	390	11.6	3,048	584,150	18.1
80+	535	2.8	248	1.3	783	4.1	42	11.9	28	6.4	208	6.2	1,061	240,544	7.4
Total	10,004		9,203		19,207		352		436		3,348		23,343	3,235,797	
**Symptoms**															

No	516	17.3	1,509	34.2	2,025	27.4	2	33.3	3	9.7	125	18.6	2,155	40,201	18.3
Yes	2,465	82.7	2,900	65.8	5,365	72.6	4	66.7	28	90.3	547	81.4	5,944	179,124	81.7
Total	2,981		4,409		7,390		6		31		672		8,099	219,325	
**Precondition (any)**															
No	1,726	17.3	8,882	96.5	10,608	55.2	254	72.2	89	20.4	369	11.0	11,320	715,001	22.1
Yes	8,278	82.7	321	3.5	8,599	44.8	98	27.8	347	79.6	2,979	89.0	12,023	2,521,091	77.9
Total	10,004		9,203		19,207		352		436		3,348		23,343	3,236,092	
**Hospitalisation**															
No	7,751	89.2	104	80.0	7,855	89.0	272	80.0	309	80.7	2,399	92.5	10,835	2,935,725	92.5
Yes	940	10.8	26	20.0	966	11.0	68	20.0	74	19.3	195	7.5	1,303	238,865	7.5
Total	8,691		130		8,821		340		383		2,594		12,138	3,174,590	
**ICU admission**															
No	8,489	98.6	104	100.0	8,593	98.6	332	97.9	380	97.7	2,553	99.4	11,858	3,150,994	99.0
Yes	121	1.4	0	0.0	121	1.4	7	2.1	9	2.3	16	0.6	153	33,219	1.0
Total	8,610		104		8,714		339		389		2,569		12,011	3,184,213	
**Mortality/outcome**															
Alive/on treatment	7,430	98.0	60	98.4	7,490	98.0	295	96.1	309	94.8	1,773	96.0	9,867	3,009,745	97.8
Died	154	2.0	1	1.6	155	2.0	12	3.9	17	5.2	73	4.0	257	67,395	2.2
Total	7,584		61		7,645		307		326		1,846		10,124	3,077,140	
**Cases imported**															
No	6,120	98.7	23	63.9	6,143	98.5	263	98.5	107	91.5	694	99.6	7,207	2,557,527	99.5
Yes	80	1.3	13	36.1	93	1.5	4	1.5	10	8.5	3	0.4	110	13,991	0.5
Total	6,200		36		6,236		267		117		697		7,317	2,571,518	
**Healthcare worker**															
No	7,276	93.7	4,709	95.2	11,985	94.3	85	80.2	358	92.7	2,425	92.0	14,853	2,139,314	94.8
Yes	491	6.3	239	4.8	730	5.7	21	19.8	28	7.3	211	8.0	990	117,974	5.2
Total	7,767		4,948		12,715		106		386		2,636		15,843	2,257,288	

SGTF: S gene target failure; VOC: variant of concern
